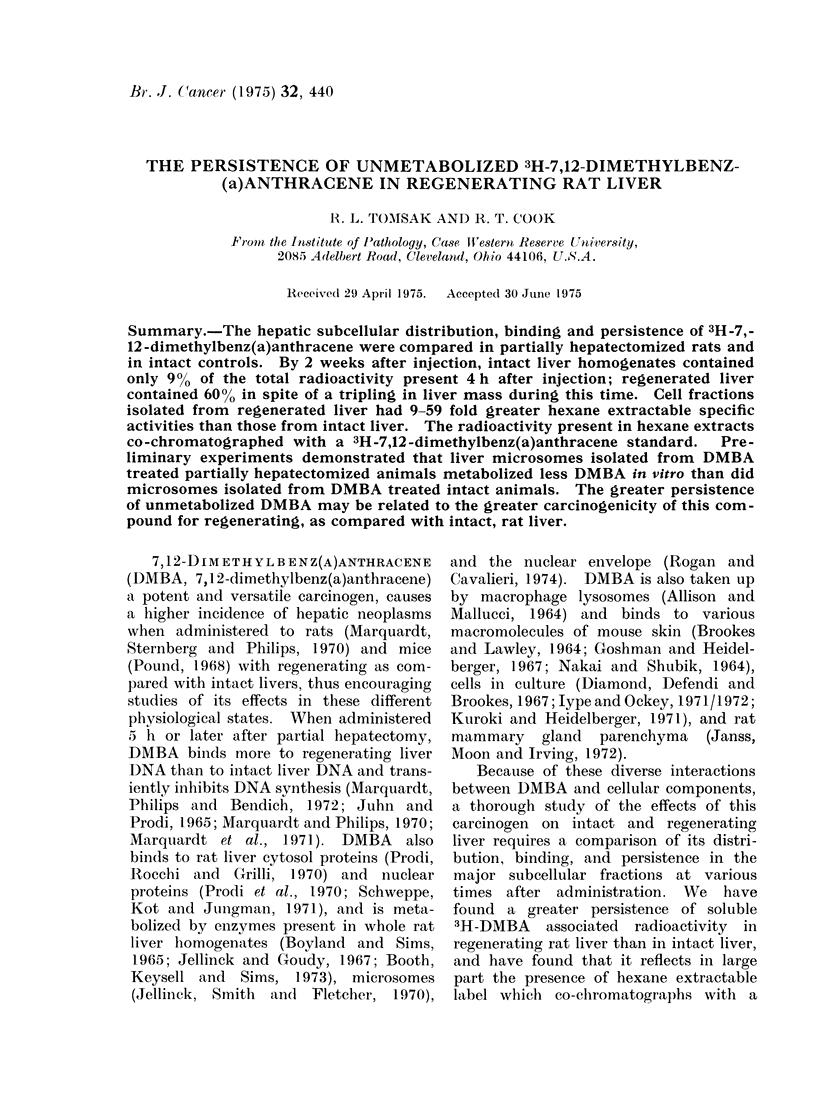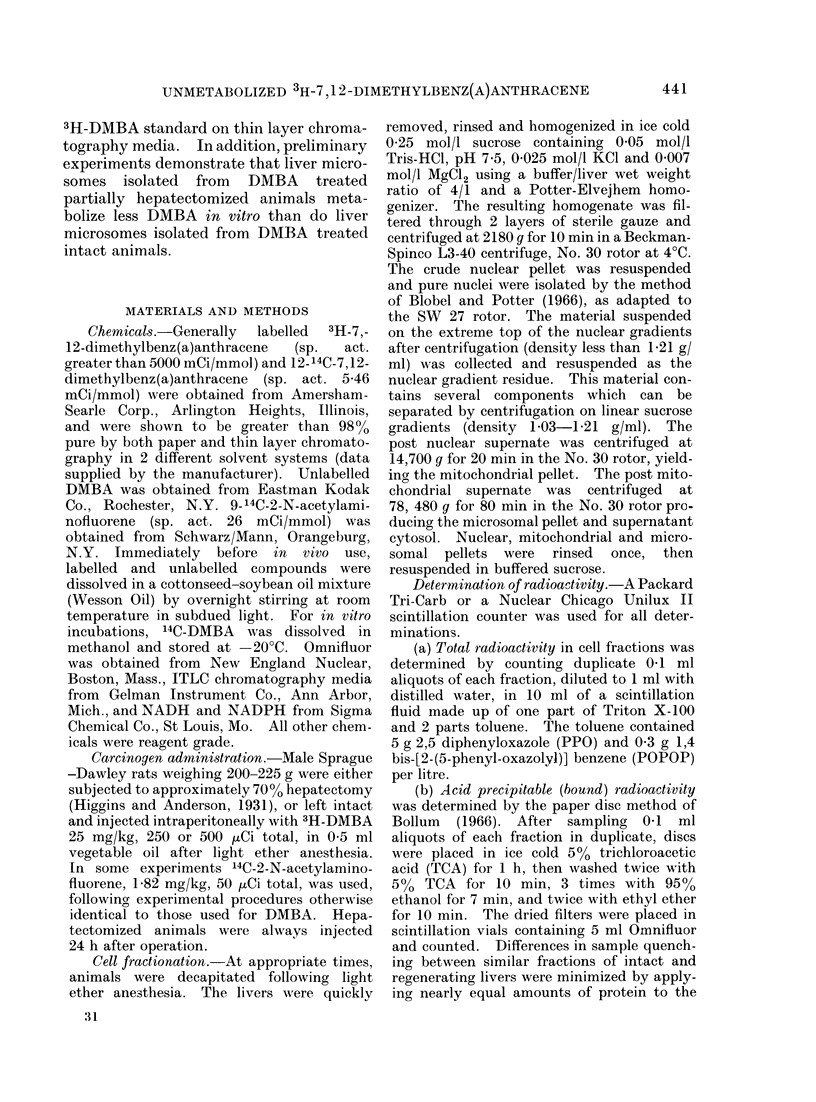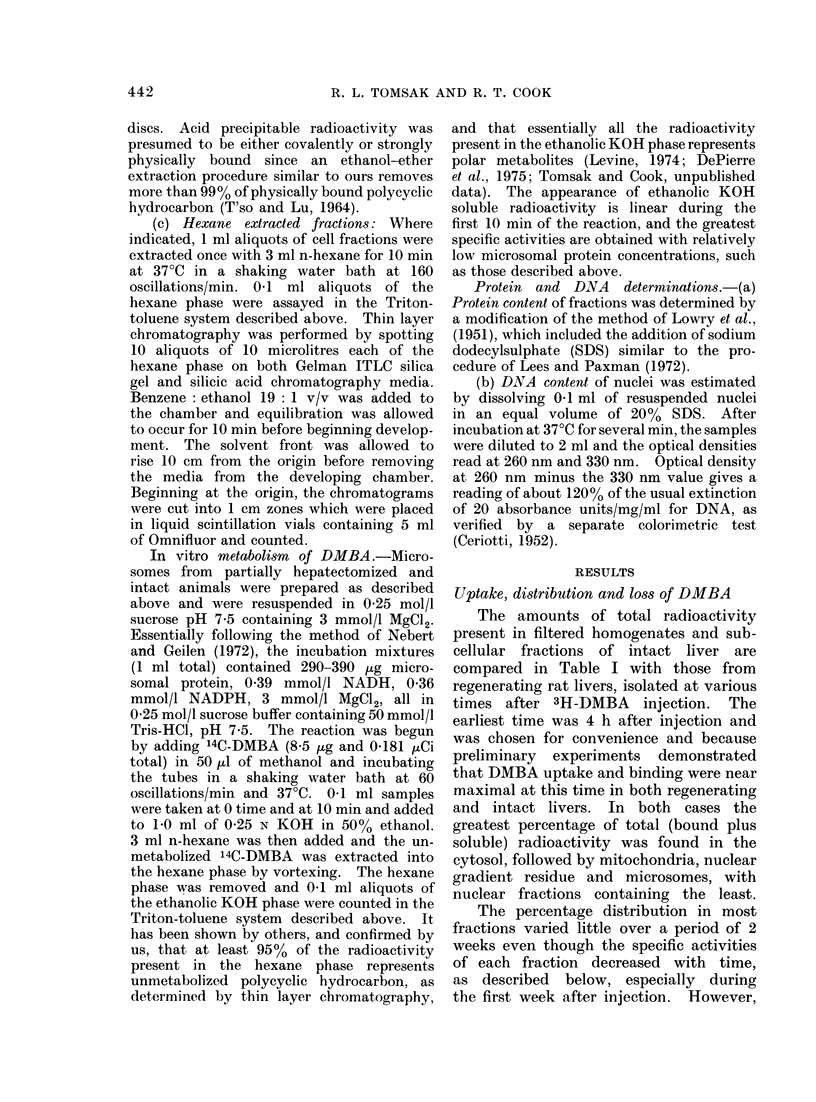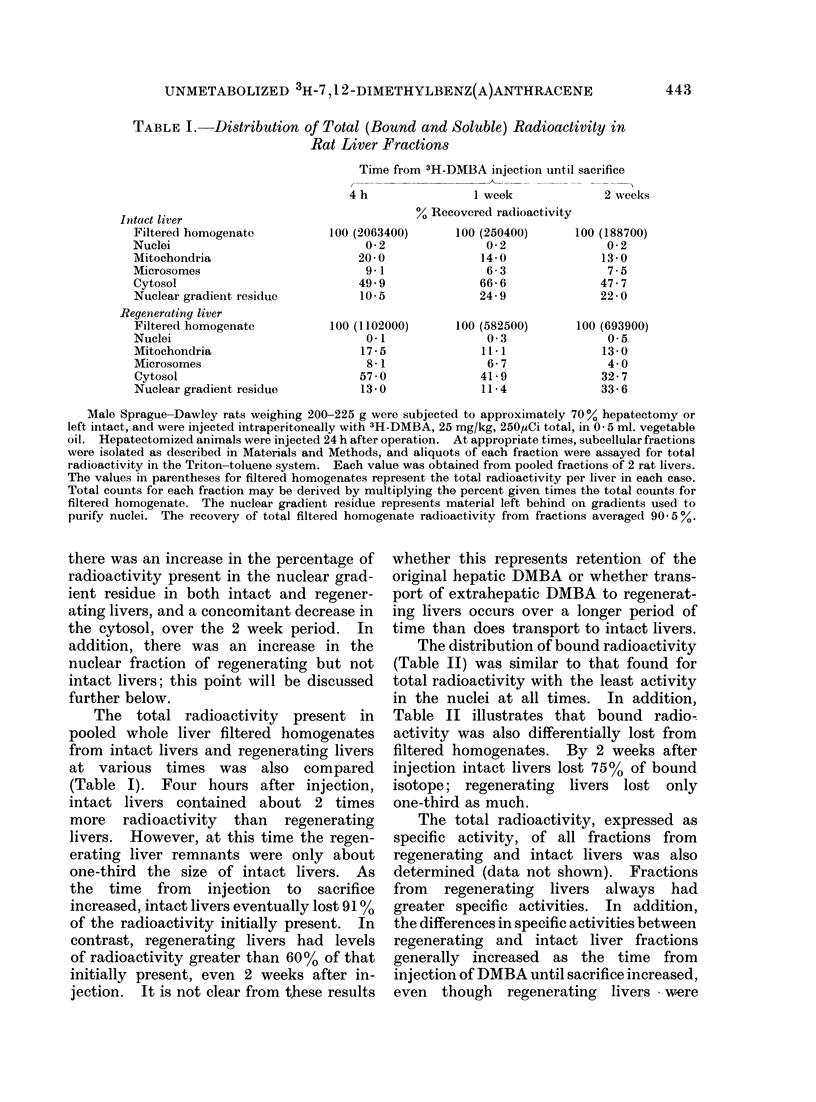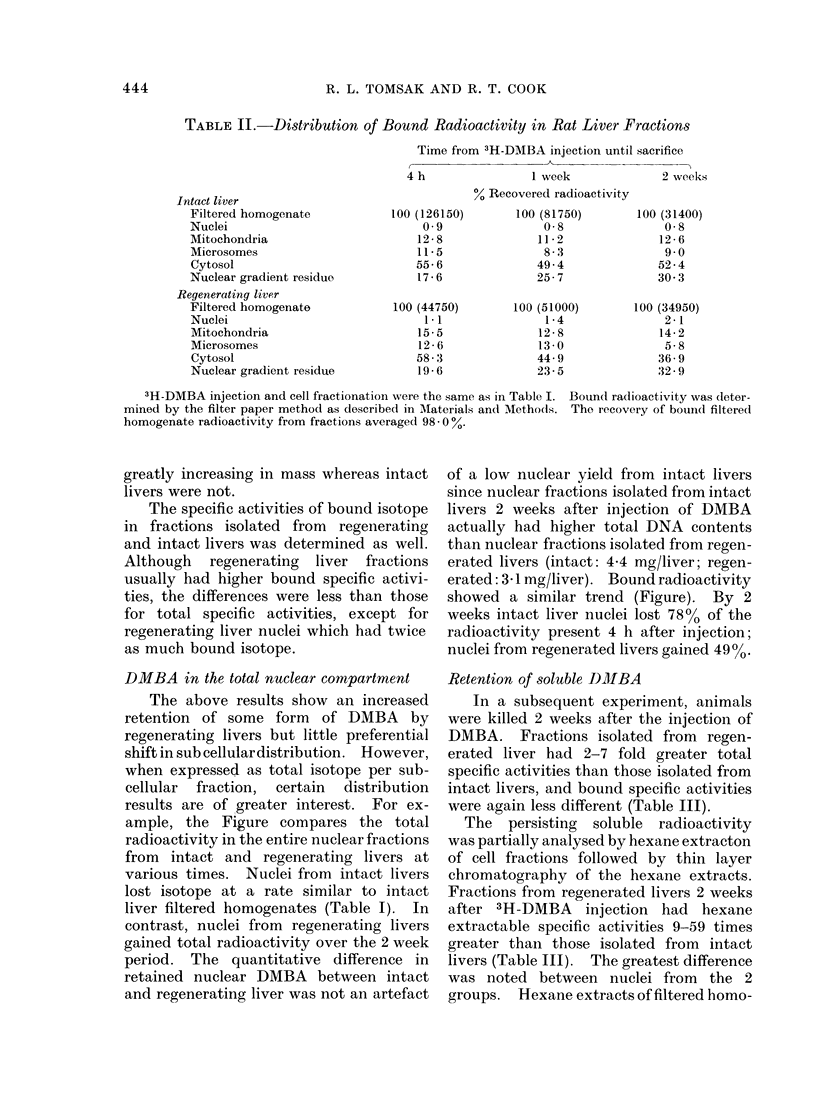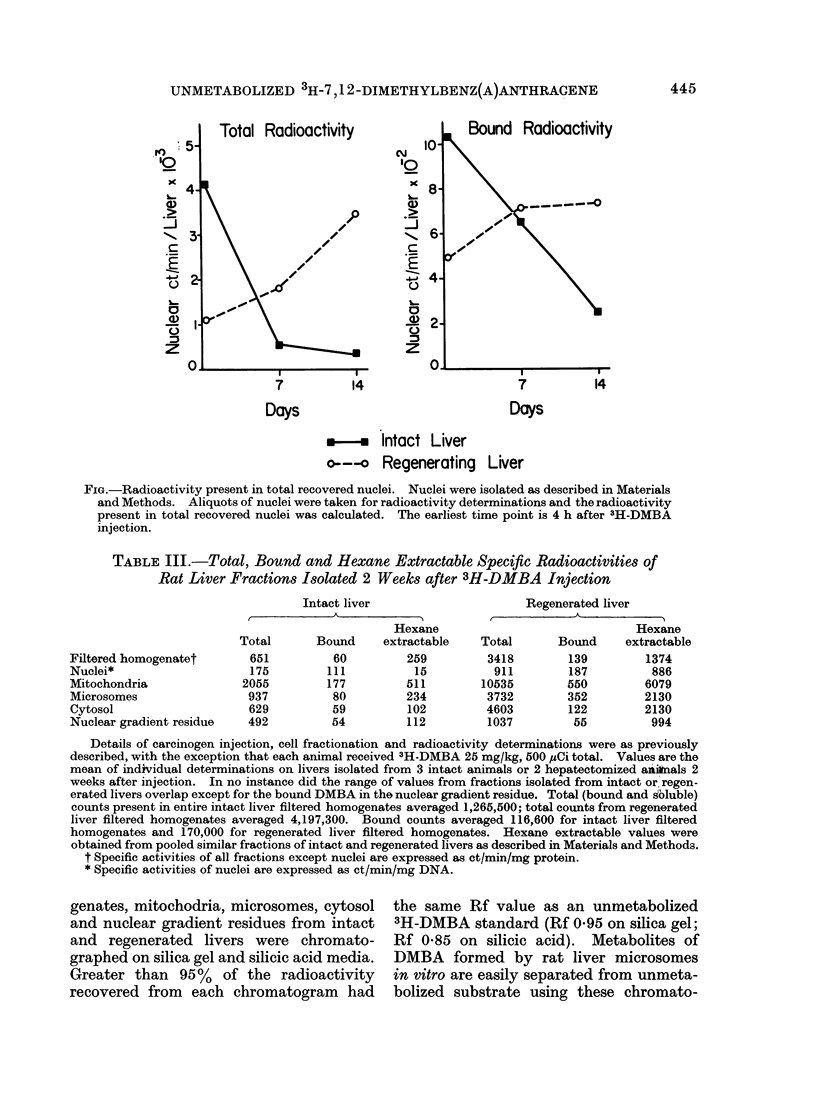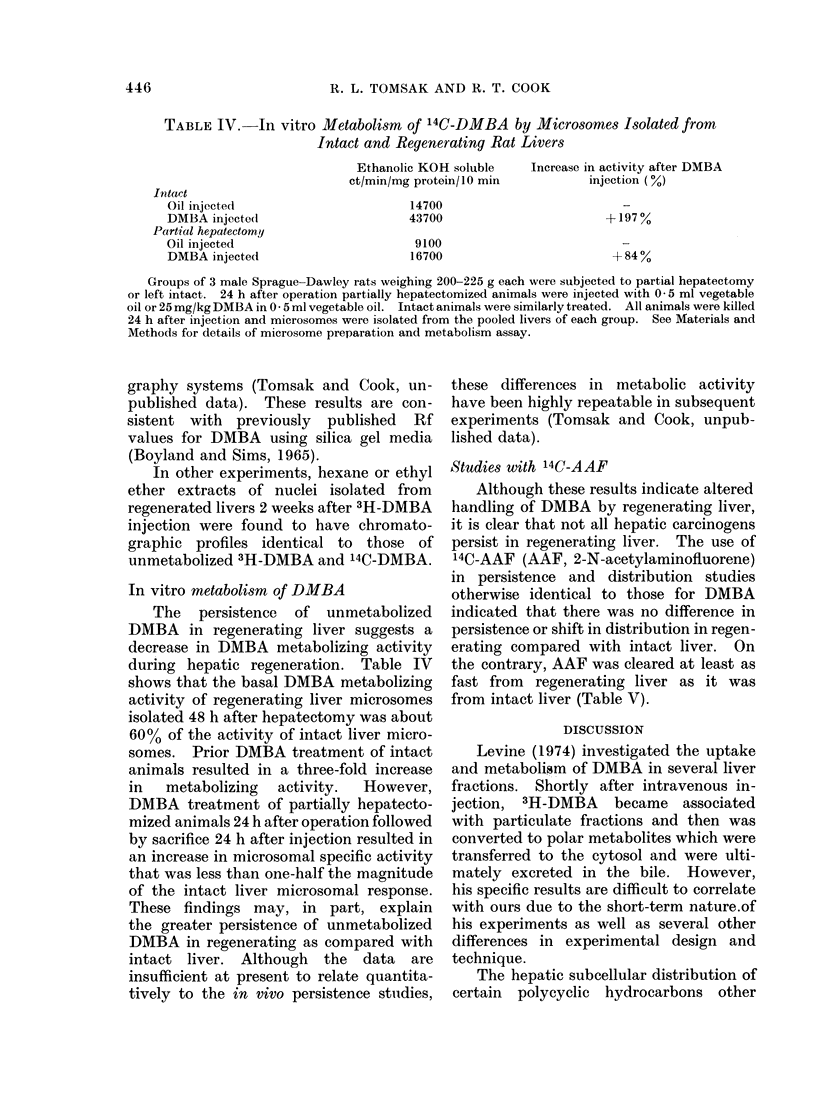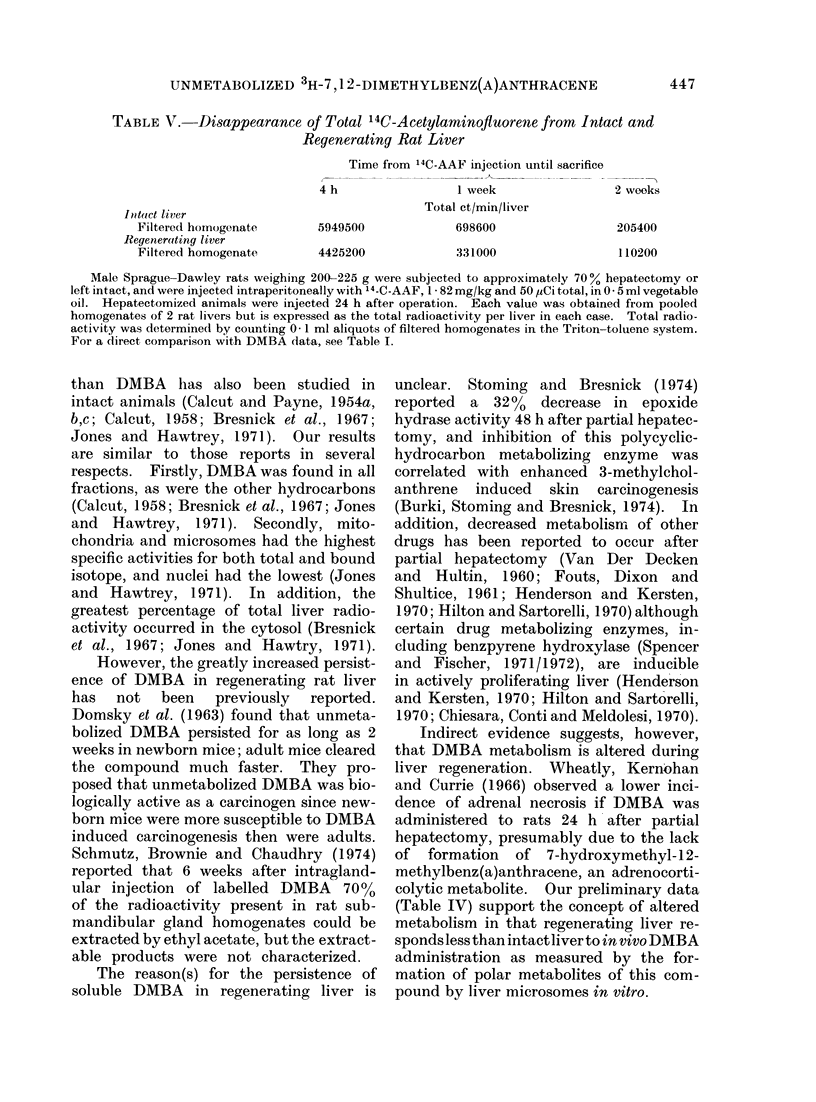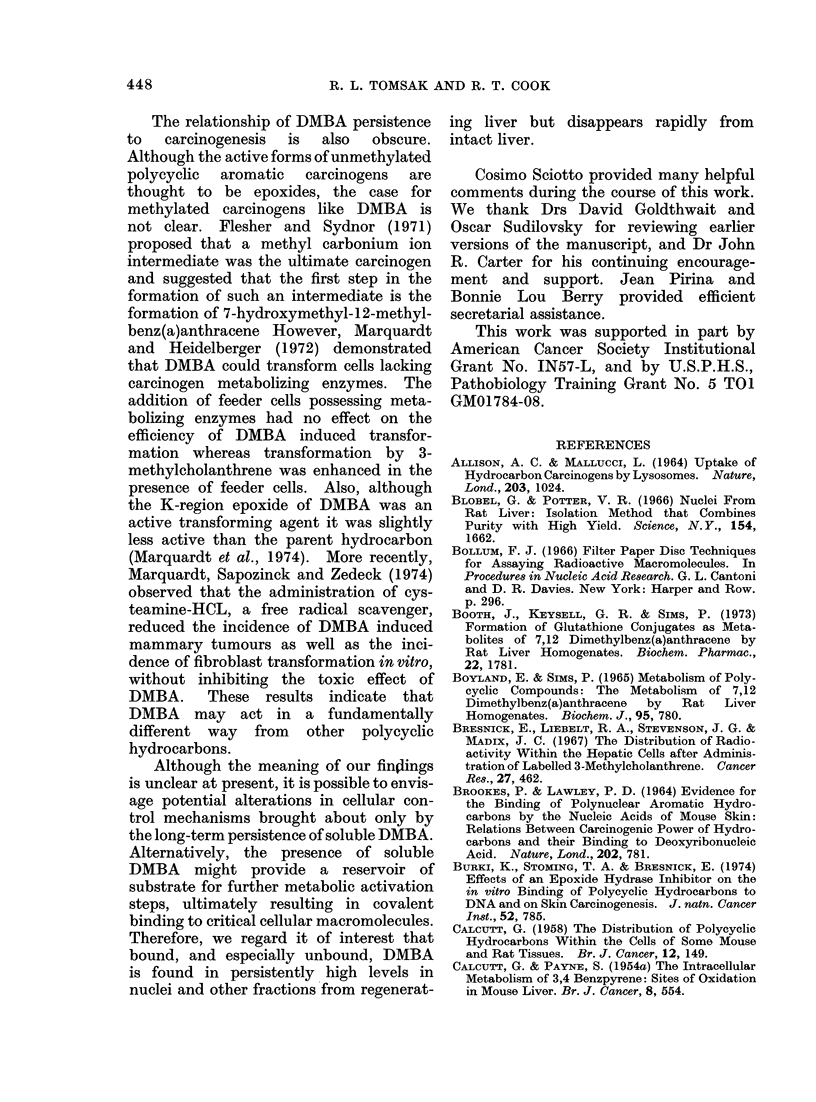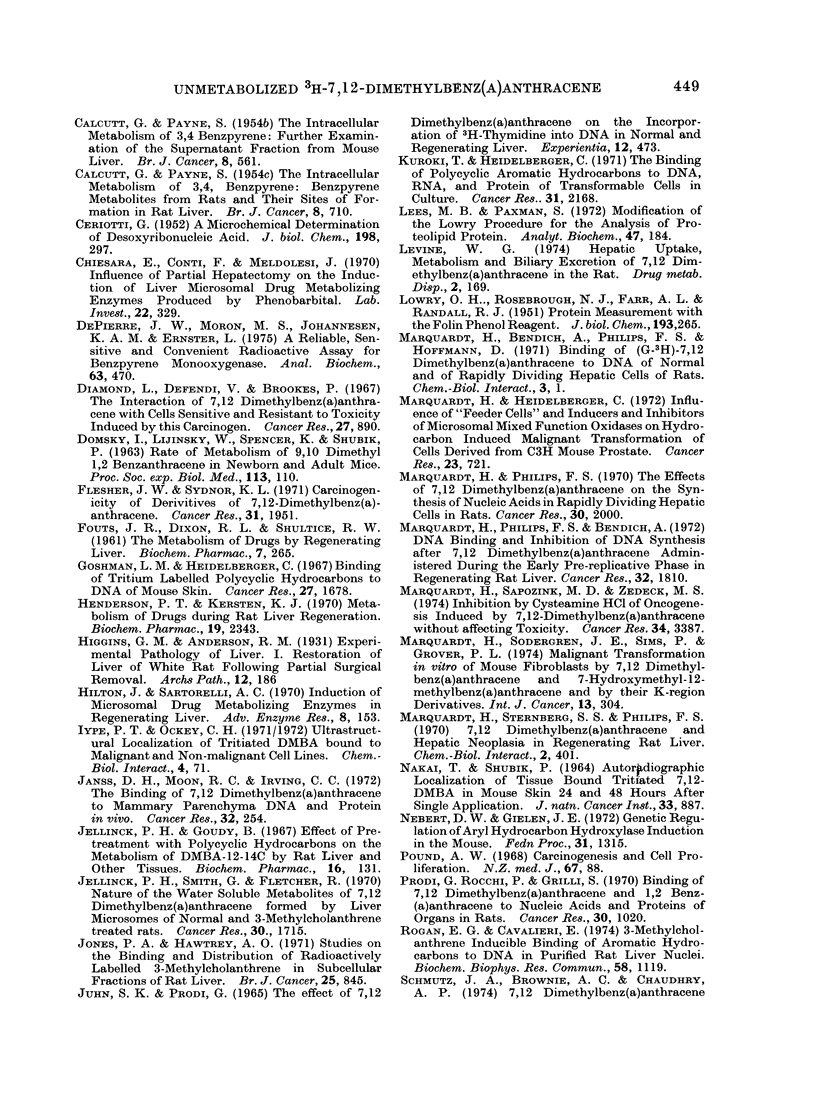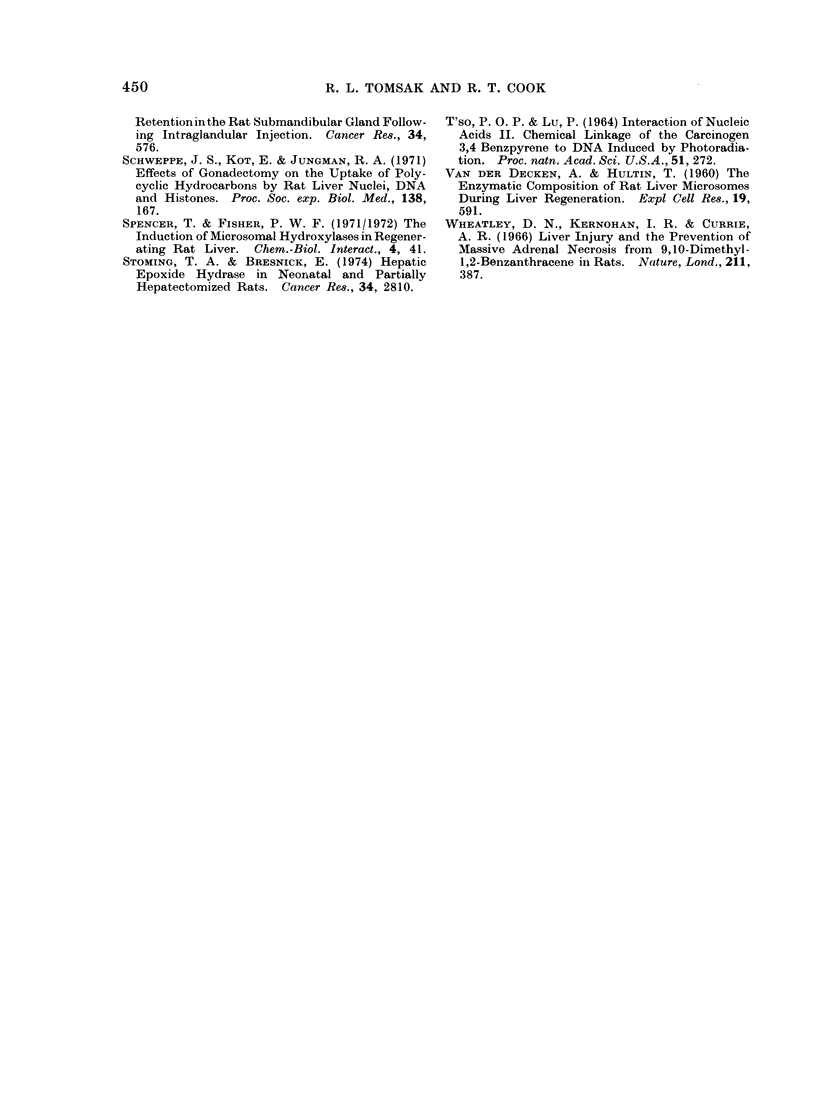# The persistence of unmetabolized 3H-7,12-dimethylbenz(a)anthracene in regenerating rat liver.

**DOI:** 10.1038/bjc.1975.245

**Published:** 1975-10

**Authors:** R. L. Tomsak, R. T. Cook

## Abstract

The hepatic subcellular distribution, binding and persistence of 3H-7,12-dimethylbenz(a)anthracene were compared in partially hepatectomized rats and in intact controls. By 2 weeks after injection, intact liver homogenates contained only 9% of the total radioactivity present 4 h after injection; regenerated liver contained 60% in spite of a tripling in liver mass during this time. Cell fractions isolated from regenerated liver had 9-59 fold greater hexane extractable specific activities than those from intact liver. The radioactivity present in hexane extracts co-chromatographed with a 3H-7,12-dimethylbenz(a)anthracene standard. Preliminary experiments demonstrated that liver microsomes isolated from DMBA treated partially hepatectomized animals metabolized less DMBA in vitro than did microsomes isolated from DMBA treated intact animals. The greater persistence of unmetabolized DMBA may be related to the greater carcinogenicity of this compound for regenerating, as compared with intact, rat liver.


*Br. J. Cancer* (1975) **32**, 440

### THE PERSISTENCE OF UNMETABOLIZED $^{3}$H-7,12-DIMETHYLBENZ-(a)ANTHRACENE IN REGENERATING RAT LIVER

R. L. TOMSAK AND R. T. COOK

*From the Institute of Pathology, Case Western Reserve University, 2085 Adelbert Road, Cleveland, Ohio* 44106, *U.S.A.*

Received 29 April 1975. Accepted 30 June 1975

**Summary.—The hepatic subcellular distribution, binding and persistence of $^{3}$H-7,-12-dimethylbenz(a)anthracene were compared in partially hepatectomized rats and in intact controls. By 2 weeks after injection, intact liver homogenates contained only 9% of the total radioactivity present 4 h after injection; regenerated liver contained 60% in spite of a tripling in liver mass during this time. Cell fractions isolated from regenerated liver had 9–59 fold greater hexane extractable specific activities than those from intact liver. The radioactivity present in hexane extracts co-chromatographed with a $^{3}$H-7,12-dimethylbenz(a)anthracene standard. Preliminary experiments demonstrated that liver microsomes isolated from DMBA treated partially hepatectomized animals metabolized less DMBA *in vitro* than did microsomes isolated from DMBA treated intact animals. The greater persistence of unmetabolized DMBA may be related to the greater carcinogenicity of this compound for regenerating, as compared with intact, rat liver.**

7,12-DIMETHYLBENZ(A)ANTHRACENE (DMBA, 7,12-dimethylbenz(a)anthracene) a potent and versatile carcinogen, causes a higher incidence of hepatic neoplasms when administered to rats (Marquardt, Sternberg and Philips, 1970) and mice (Pound, 1968) with regenerating as compared with intact livers, thus encouraging studies of its effects in these different physiological states. When administered 5 h or later after partial hepatectomy, DMBA binds more to regenerating liver DNA than to intact liver DNA and transiently inhibits DNA synthesis (Marquardt, Philips and Bendich, 1972; Juhn and Prodi, 1965; Marquardt and Philips, 1970; Marquardt *et al.*, 1971). DMBA also binds to rat liver cytosol proteins (Prodi, Rocchi and Grilli, 1970) and nuclear proteins (Prodi *et al.*, 1970; Schweppe, Kot and Jungman, 1971), and is metabolized by enzymes present in whole rat liver homogenates (Boyland and Sims, 1965; Jellinck and Goudy, 1967; Booth, Keysell and Sims, 1973), microsomes (Jellinck, Smith and Fletcher, 1970), and the nuclear envelope (Rogan and Cavalieri, 1974). DMBA is also taken up by macrophage lysosomes (Allison and Mallucci, 1964) and binds to various macromolecules of mouse skin (Brookes and Lawley, 1964; Goshman and Heidelberger, 1967; Nakai and Shubik, 1964), cells in culture (Diamond, Defendi and Brookes, 1967; Iype and Ockey, 1971/1972; Kuroki and Heidelberger, 1971), and rat mammary gland parenchyma (Janss, Moon and Irving, 1972).

Because of these diverse interactions between DMBA and cellular components, a thorough study of the effects of this carcinogen on intact and regenerating liver requires a comparison of its distribution, binding, and persistence in the major subcellular fractions at various times after administration. We have found a greater persistence of soluble $^{3}$H-DMBA associated radioactivity in regenerating rat liver than in intact liver, and have found that it reflects in large part the presence of hexane extractable label which co-chromatographs with a

$^{3}$H-DMBA standard on thin layer chromatography media. In addition, preliminary experiments demonstrate that liver microsomes isolated from DMBA treated partially hepatectomized animals metabolize less DMBA *in vitro* than do liver microsomes isolated from DMBA treated intact animals.

### MATERIALS AND METHODS

*Chemicals.*—Generally labelled $^{3}$H-7,-12-dimethylbenz(a)anthracene (sp. act. greater than 5000 mCi/mmol) and 12-$^{14}$C-7,12-dimethylbenz(a)anthracene (sp. act. 5·46 mCi/mmol) were obtained from Amersham-Searle Corp., Arlington Heights, Illinois, and were shown to be greater than 98% pure by both paper and thin layer chromatography in 2 different solvent systems (data supplied by the manufacturer). Unlabelled DMBA was obtained from Eastman Kodak Co., Rochester, N.Y. 9-$^{14}$C-2-N-acetylaminofluorene (sp. act. 26 mCi/mmol) was obtained from Schwarz/Mann, Orangeburg, N.Y. Immediately before *in vivo* use, labelled and unlabelled compounds were dissolved in a cottonseed–soybean oil mixture (Wesson Oil) by overnight stirring at room temperature in subdued light. For *in vitro* incubations, $^{14}$C-DMBA was dissolved in methanol and stored at −20°C. Omnifluor was obtained from New England Nuclear, Boston, Mass., ITLC chromatography media from Gelman Instrument Co., Ann Arbor, Mich., and NADH and NADPH from Sigma Chemical Co., St Louis, Mo. All other chemicals were reagent grade.

*Carcinogen administration.*—Male Sprague–Dawley rats weighing 200–225 g were either subjected to approximately 70% hepatectomy (Higgins and Anderson, 1931), or left intact and injected intraperitoneally with $^{3}$H-DMBA 25 mg/kg, 250 or 500 $\mu$Ci total, in 0·5 ml vegetable oil after light ether anesthesia. In some experiments $^{14}$C-2-N-acetylaminofluorene, 1·82 mg/kg, 50 $\mu$Ci total, was used, following experimental procedures otherwise identical to those used for DMBA. Hepatectomized animals were always injected 24 h after operation.

*Cell fractionation.*—At appropriate times, animals were decapitated following light ether anesthesia. The livers were quickly removed, rinsed and homogenized in ice cold 0·25 mol/l sucrose containing 0·05 mol/l Tris-HCl, pH 7·5, 0·025 mol/l KCl and 0·007 mol/l $MgCl_2$ using a buffer/liver wet weight ratio of 4/1 and a Potter-Elvejhem homogenizer. The resulting homogenate was filtered through 2 layers of sterile gauze and centrifuged at 2180 *g* for 10 min in a Beckman-Spinco L3-40 centrifuge, No. 30 rotor at 4°C. The crude nuclear pellet was resuspended and pure nuclei were isolated by the method of Blobel and Potter (1966), as adapted to the SW 27 rotor. The material suspended on the extreme top of the nuclear gradients after centrifugation (density less than 1·21 g/ml) was collected and resuspended as the nuclear gradient residue. This material contains several components which can be separated by centrifugation on linear sucrose gradients (density 1·03—1·21 g/ml). The post nuclear supernate was centrifuged at 14,700 *g* for 20 min in the No. 30 rotor, yielding the mitochondrial pellet. The post mitochondrial supernate was centrifuged at 78, 480 *g* for 80 min in the No. 30 rotor producing the microsomal pellet and supernatant cytosol. Nuclear, mitochondrial and microsomal pellets were rinsed once, then resuspended in buffered sucrose.

*Determination of radioactivity.*—A Packard Tri-Carb or a Nuclear Chicago Unilux II scintillation counter was used for all determinations.

(a) *Total radioactivity* in cell fractions was determined by counting duplicate 0·1 ml aliquots of each fraction, diluted to 1 ml with distilled water, in 10 ml of a scintillation fluid made up of one part of Triton X-100 and 2 parts toluene. The toluene contained 5 g 2,5 diphenyloxazole (PPO) and 0·3 g 1,4 bis-[2-(5-phenyl-oxazolyl)] benzene (POPOP) per litre.

(b) *Acid precipitable* (*bound*) *radioactivity* was determined by the paper disc method of Bollum (1966). After sampling 0·1 ml aliquots of each fraction in duplicate, discs were placed in ice cold 5% trichloroacetic acid (TCA) for 1 h, then washed twice with 5% TCA for 10 min, 3 times with 95% ethanol for 7 min, and twice with ethyl ether for 10 min. The dried filters were placed in scintillation vials containing 5 ml Omnifluor and counted. Differences in sample quenching between similar fractions of intact and regenerating livers were minimized by applying nearly equal amounts of protein to the discs. Acid precipitable radioactivity was presumed to be either covalently or strongly physically bound since an ethanol–ether extraction procedure similar to ours removes more than 99% of physically bound polycyclic hydrocarbon (T'so and Lu, 1964).

(c) *Hexane extracted fractions:* Where indicated, 1 ml aliquots of cell fractions were extracted once with 3 ml n-hexane for 10 min at 37°C in a shaking water bath at 160 oscillations/min. 0·1 ml aliquots of the hexane phase were assayed in the Triton-toluene system described above. Thin layer chromatography was performed by spotting 10 aliquots of 10 microlitres each of the hexane phase on both Gelman ITLC silica gel and silicic acid chromatography media. Benzene : ethanol 19 : 1 v/v was added to the chamber and equilibration was allowed to occur for 10 min before beginning development. The solvent front was allowed to rise 10 cm from the origin before removing the media from the developing chamber. Beginning at the origin, the chromatograms were cut into 1 cm zones which were placed in liquid scintillation vials containing 5 ml of Omnifluor and counted.

In vitro *metabolism of DMBA*.—Microsomes from partially hepatectomized and intact animals were prepared as described above and were resuspended in 0·25 mol/l sucrose pH 7·5 containing 3 mmol/l $MgCl_2$. Essentially following the method of Nebert and Geilen (1972), the incubation mixtures (1 ml total) contained 290–390 $\mu$g microsomal protein, 0·39 mmol/l NADH, 0·36 mmol/l NADPH, 3 mmol/l $MgCl_2$, all in 0·25 mol/l sucrose buffer containing 50 mmol/l Tris-HCl, pH 7·5. The reaction was begun by adding $^{14}$C-DMBA (8·5 $\mu$g and 0·181 $\mu$Ci total) in 50 $\mu$l of methanol and incubating the tubes in a shaking water bath at 60 oscillations/min and 37°C. 0·1 ml samples were taken at 0 time and at 10 min and added to 1·0 ml of 0·25 N KOH in 50% ethanol. 3 ml n-hexane was then added and the unmetabolized $^{14}$C-DMBA was extracted into the hexane phase by vortexing. The hexane phase was removed and 0·1 ml aliquots of the ethanolic KOH phase were counted in the Triton-toluene system described above. It has been shown by others, and confirmed by us, that at least 95% of the radioactivity present in the hexane phase represents unmetabolized polycyclic hydrocarbon, as determined by thin layer chromatography, and that essentially all the radioactivity present in the ethanolic KOH phase represents polar metabolites (Levine, 1974; DePierre *et al.*, 1975; Tomsak and Cook, unpublished data). The appearance of ethanolic KOH soluble radioactivity is linear during the first 10 min of the reaction, and the greatest specific activities are obtained with relatively low microsomal protein concentrations, such as those described above.

*Protein and DNA determinations*.—(a) *Protein content* of fractions was determined by a modification of the method of Lowry *et al.*, (1951), which included the addition of sodium dodecylsulphate (SDS) similar to the procedure of Lees and Paxman (1972).

(b) *DNA content* of nuclei was estimated by dissolving 0·1 ml of resuspended nuclei in an equal volume of 20% SDS. After incubation at 37°C for several min, the samples were diluted to 2 ml and the optical densities read at 260 nm and 330 nm. Optical density at 260 nm minus the 330 nm value gives a reading of about 120% of the usual extinction of 20 absorbance units/mg/ml for DNA, as verified by a separate colorimetric test (Ceriotti, 1952).

### RESULTS

### *Uptake, distribution and loss of DMBA*

The amounts of total radioactivity present in filtered homogenates and subcellular fractions of intact liver are compared in Table I with those from regenerating rat livers, isolated at various times after $^{3}$H-DMBA injection. The earliest time was 4 h after injection and was chosen for convenience and because preliminary experiments demonstrated that DMBA uptake and binding were near maximal at this time in both regenerating and intact livers. In both cases the greatest percentage of total (bound plus soluble) radioactivity was found in the cytosol, followed by mitochondria, nuclear gradient residue and microsomes, with nuclear fractions containing the least.

The percentage distribution in most fractions varied little over a period of 2 weeks even though the specific activities of each fraction decreased with time, as described below, especially during the first week after injection. However,

TABLE I.—*Distribution of Total (Bound and Soluble) Radioactivity in Rat Liver Fractions*

| | Time from $^3$H-DMBA injection until sacrifice | | |
|---|---|---|---|
| | 4 h | 1 week | 2 weeks |
| | % Recovered radioactivity | | |
| *Intact liver* | | | |
| Filtered homogenate | 100 (2063400) | 100 (250400) | 100 (188700) |
| Nuclei | 0·2 | 0·2 | 0·2 |
| Mitochondria | 20·0 | 14·0 | 13·0 |
| Microsomes | 9·1 | 6·3 | 7·5 |
| Cytosol | 49·9 | 66·6 | 47·7 |
| Nuclear gradient residue | 10·5 | 24·9 | 22·0 |
| *Regenerating liver* | | | |
| Filtered homogenate | 100 (1102000) | 100 (582500) | 100 (693900) |
| Nuclei | 0·1 | 0·3 | 0·5 |
| Mitochondria | 17·5 | 11·1 | 13·0 |
| Microsomes | 8·1 | 6·7 | 4·0 |
| Cytosol | 57·0 | 41·9 | 32·7 |
| Nuclear gradient residue | 13·0 | 11·4 | 33·6 |

Male Sprague–Dawley rats weighing 200–225 g were subjected to approximately 70% hepatectomy or left intact, and were injected intraperitoneally with $^3$H-DMBA, 25 mg/kg, 250$\mu$Ci total, in 0·5 ml. vegetable oil. Hepatectomized animals were injected 24 h after operation. At appropriate times, subcellular fractions were isolated as described in Materials and Methods, and aliquots of each fraction were assayed for total radioactivity in the Triton–toluene system. Each value was obtained from pooled fractions of 2 rat livers. The values in parentheses for filtered homogenates represent the total radioactivity per liver in each case. Total counts for each fraction may be derived by multiplying the percent given times the total counts for filtered homogenate. The nuclear gradient residue represents material left behind on gradients used to purify nuclei. The recovery of total filtered homogenate radioactivity from fractions averaged 90·5%.

there was an increase in the percentage of radioactivity present in the nuclear gradient residue in both intact and regenerating livers, and a concomitant decrease in the cytosol, over the 2 week period. In addition, there was an increase in the nuclear fraction of regenerating but not intact livers; this point will be discussed further below.

The total radioactivity present in pooled whole liver filtered homogenates from intact livers and regenerating livers at various times was also compared (Table I). Four hours after injection, intact livers contained about 2 times more radioactivity than regenerating livers. However, at this time the regenerating liver remnants were only about one-third the size of intact livers. As the time from injection to sacrifice increased, intact livers eventually lost 91% of the radioactivity initially present. In contrast, regenerating livers had levels of radioactivity greater than 60% of that initially present, even 2 weeks after injection. It is not clear from these results whether this represents retention of the original hepatic DMBA or whether transport of extrahepatic DMBA to regenerating livers occurs over a longer period of time than does transport to intact livers.

The distribution of bound radioactivity (Table II) was similar to that found for total radioactivity with the least activity in the nuclei at all times. In addition, Table II illustrates that bound radioactivity was also differentially lost from filtered homogenates. By 2 weeks after injection intact livers lost 75% of bound isotope; regenerating livers lost only one-third as much.

The total radioactivity, expressed as specific activity, of all fractions from regenerating and intact livers was also determined (data not shown). Fractions from regenerating livers always had greater specific activities. In addition, the differences in specific activities between regenerating and intact liver fractions generally increased as the time from injection of DMBA until sacrifice increased, even though regenerating livers were

TABLE II.—*Distribution of Bound Radioactivity in Rat Liver Fractions*

| | Time from $^3$H-DMBA injection until sacrifice | | |
|---|---|---|---|
| | 4 h | 1 week | 2 weeks |
| | % Recovered radioactivity | | |
| *Intact liver* | | | |
| Filtered homogenate | 100 (126150) | 100 (81750) | 100 (31400) |
| Nuclei | 0·9 | 0·8 | 0·8 |
| Mitochondria | 12·8 | 11·2 | 12·6 |
| Microsomes | 11·5 | 8·3 | 9·0 |
| Cytosol | 55·6 | 49·4 | 52·4 |
| Nuclear gradient residue | 17·6 | 25·7 | 30·3 |
| *Regenerating liver* | | | |
| Filtered homogenate | 100 (44750) | 100 (51000) | 100 (34950) |
| Nuclei | 1·1 | 1·4 | 2·1 |
| Mitochondria | 15·5 | 12·8 | 14·2 |
| Microsomes | 12·6 | 13·0 | 5·8 |
| Cytosol | 58·3 | 44·9 | 36·9 |
| Nuclear gradient residue | 19·6 | 23·5 | 32·9 |

$^3$H-DMBA injection and cell fractionation were the same as in Table I. Bound radioactivity was determined by the filter paper method as described in Materials and Methods. The recovery of bound filtered homogenate radioactivity from fractions averaged 98·0%.

greatly increasing in mass whereas intact livers were not.

The specific activities of bound isotope in fractions isolated from regenerating and intact livers was determined as well. Although regenerating liver fractions usually had higher bound specific activities, the differences were less than those for total specific activities, except for regenerating liver nuclei which had twice as much bound isotope.

*DMBA in the total nuclear compartment*

The above results show an increased retention of some form of DMBA by regenerating livers but little preferential shift in subcellular distribution. However, when expressed as total isotope per subcellular fraction, certain distribution results are of greater interest. For example, the Figure compares the total radioactivity in the entire nuclear fractions from intact and regenerating livers at various times. Nuclei from intact livers lost isotope at a rate similar to intact liver filtered homogenates (Table I). In contrast, nuclei from regenerating livers gained total radioactivity over the 2 week period. The quantitative difference in retained nuclear DMBA between intact and regenerating liver was not an artefact of a low nuclear yield from intact livers since nuclear fractions isolated from intact livers 2 weeks after injection of DMBA actually had higher total DNA contents than nuclear fractions isolated from regenerated livers (intact: 4·4 mg/liver; regenerated: 3·1 mg/liver). Bound radioactivity showed a similar trend (Figure). By 2 weeks intact liver nuclei lost 78% of the radioactivity present 4 h after injection; nuclei from regenerated livers gained 49%.

*Retention of soluble DMBA*

In a subsequent experiment, animals were killed 2 weeks after the injection of DMBA. Fractions isolated from regenerated liver had 2–7 fold greater total specific activities than those isolated from intact livers, and bound specific activities were again less different (Table III).

The persisting soluble radioactivity was partially analysed by hexane extracton of cell fractions followed by thin layer chromatography of the hexane extracts. Fractions from regenerated livers 2 weeks after $^3$H-DMBA injection had hexane extractable specific activities 9–59 times greater than those isolated from intact livers (Table III). The greatest difference was noted between nuclei from the 2 groups. Hexane extracts of filtered homo-

FIG.—Radioactivity present in total recovered nuclei. Nuclei were isolated as described in Materials and Methods. Aliquots of nuclei were taken for radioactivity determinations and the radioactivity present in total recovered nuclei was calculated. The earliest time point is 4 h after $^3$H-DMBA injection.

TABLE III.—*Total, Bound and Hexane Extractable Specific Radioactivities of Rat Liver Fractions Isolated 2 Weeks after $^3$H-DMBA Injection*

| | Intact liver | | | Regenerated liver | | |
|---|---|---|---|---|---|---|
| | Total | Bound | Hexane extractable | Total | Bound | Hexane extractable |
| Filtered homogenate† | 651 | 60 | 259 | 3418 | 139 | 1374 |
| Nuclei* | 175 | 111 | 15 | 911 | 187 | 886 |
| Mitochondria | 2055 | 177 | 511 | 10535 | 550 | 6079 |
| Microsomes | 937 | 80 | 234 | 3732 | 352 | 2130 |
| Cytosol | 629 | 59 | 102 | 4603 | 122 | 2130 |
| Nuclear gradient residue | 492 | 54 | 112 | 1037 | 55 | 994 |

Details of carcinogen injection, cell fractionation and radioactivity determinations were as previously described, with the exception that each animal received $^3$H-DMBA 25 mg/kg, 500 $\mu$Ci total. Values are the mean of individual determinations on livers isolated from 3 intact animals or 2 hepatectomized animals 2 weeks after injection. In no instance did the range of values from fractions isolated from intact or regenerated livers overlap except for the bound DMBA in the nuclear gradient residue. Total (bound and soluble) counts present in entire intact liver filtered homogenates averaged 1,265,500; total counts from regenerated liver filtered homogenates averaged 4,197,300. Bound counts averaged 116,600 for intact liver filtered homogenates and 170,000 for regenerated liver filtered homogenates. Hexane extractable values were obtained from pooled similar fractions of intact and regenerated livers as described in Materials and Methods.
† Specific activities of all fractions except nuclei are expressed as ct/min/mg protein.
* Specific activities of nuclei are expressed as ct/min/mg DNA.

genates, mitochodria, microsomes, cytosol and nuclear gradient residues from intact and regenerated livers were chromatographed on silica gel and silicic acid media. Greater than 95% of the radioactivity recovered from each chromatogram had the same Rf value as an unmetabolized $^3$H-DMBA standard (Rf 0·95 on silica gel; Rf 0·85 on silicic acid). Metabolites of DMBA formed by rat liver microsomes *in vitro* are easily separated from unmetabolized substrate using these chromato-

TABLE IV.—In vitro *Metabolism of* $^{14}$*C-DMBA by Microsomes Isolated from Intact and Regenerating Rat Livers*

| | Ethanolic KOH soluble ct/min/mg protein/10 min | Increase in activity after DMBA injection (%) |
|---|---|---|
| *Intact* | | |
| Oil injected | 14700 | – |
| DMBA injected | 43700 | +197% |
| *Partial hepatectomy* | | |
| Oil injected | 9100 | – |
| DMBA injected | 16700 | +84% |

Groups of 3 male Sprague–Dawley rats weighing 200–225 g each were subjected to partial hepatectomy or left intact. 24 h after operation partially hepatectomized animals were injected with 0·5 ml vegetable oil or 25 mg/kg DMBA in 0·5 ml vegetable oil. Intact animals were similarly treated. All animals were killed 24 h after injection and microsomes were isolated from the pooled livers of each group. See Materials and Methods for details of microsome preparation and metabolism assay.

graphy systems (Tomsak and Cook, unpublished data). These results are consistent with previously published Rf values for DMBA using silica gel media (Boyland and Sims, 1965).

In other experiments, hexane or ethyl ether extracts of nuclei isolated from regenerated livers 2 weeks after $^{3}$H-DMBA injection were found to have chromatographic profiles identical to those of unmetabolized $^{3}$H-DMBA and $^{14}$C-DMBA.

### In vitro *metabolism of DMBA*

The persistence of unmetabolized DMBA in regenerating liver suggests a decrease in DMBA metabolizing activity during hepatic regeneration. Table IV shows that the basal DMBA metabolizing activity of regenerating liver microsomes isolated 48 h after hepatectomy was about 60% of the activity of intact liver microsomes. Prior DMBA treatment of intact animals resulted in a three-fold increase in metabolizing activity. However, DMBA treatment of partially hepatectomized animals 24 h after operation followed by sacrifice 24 h after injection resulted in an increase in microsomal specific activity that was less than one-half the magnitude of the intact liver microsomal response. These findings may, in part, explain the greater persistence of unmetabolized DMBA in regenerating as compared with intact liver. Although the data are insufficient at present to relate quantitatively to the *in vivo* persistence studies, these differences in metabolic activity have been highly repeatable in subsequent experiments (Tomsak and Cook, unpublished data).

### *Studies with* $^{14}$*C-AAF*

Although these results indicate altered handling of DMBA by regenerating liver, it is clear that not all hepatic carcinogens persist in regenerating liver. The use of $^{14}$C-AAF (AAF, 2-N-acetylaminofluorene) in persistence and distribution studies otherwise identical to those for DMBA indicated that there was no difference in persistence or shift in distribution in regenerating compared with intact liver. On the contrary, AAF was cleared at least as fast from regenerating liver as it was from intact liver (Table V).

### DISCUSSION

Levine (1974) investigated the uptake and metabolism of DMBA in several liver fractions. Shortly after intravenous injection, $^{3}$H-DMBA became associated with particulate fractions and then was converted to polar metabolites which were transferred to the cytosol and were ultimately excreted in the bile. However, his specific results are difficult to correlate with ours due to the short-term nature.of his experiments as well as several other differences in experimental design and technique.

The hepatic subcellular distribution of certain polycyclic hydrocarbons other

TABLE V.—*Disappearance of Total $^{14}$C-Acetylaminofluorene from Intact and Regenerating Rat Liver*

| | Time from $^{14}$C-AAF injection until sacrifice | | |
|---|---|---|---|
| | 4 h | 1 week | 2 weeks |
| | | Total ct/min/liver | |
| *Intact liver* | | | |
| Filtered homogenate | 5949500 | 698600 | 205400 |
| *Regenerating liver* | | | |
| Filtered homogenate | 4425200 | 331000 | 110200 |

Male Sprague–Dawley rats weighing 200–225 g were subjected to approximately 70% hepatectomy or left intact, and were injected intraperitoneally with $^{14}$-C-AAF, 1·82 mg/kg and 50 μCi total, in 0·5 ml vegetable oil. Hepatectomized animals were injected 24 h after operation. Each value was obtained from pooled homogenates of 2 rat livers but is expressed as the total radioactivity per liver in each case. Total radioactivity was determined by counting 0·1 ml aliquots of filtered homogenates in the Triton–toluene system. For a direct comparison with DMBA data, see Table I.

than DMBA has also been studied in intact animals (Calcut and Payne, 1954*a*, *b*,*c*; Calcut, 1958; Bresnick *et al.*, 1967; Jones and Hawtrey, 1971). Our results are similar to those reports in several respects. Firstly, DMBA was found in all fractions, as were the other hydrocarbons (Calcut, 1958; Bresnick *et al.*, 1967; Jones and Hawtrey, 1971). Secondly, mitochondria and microsomes had the highest specific activities for both total and bound isotope, and nuclei had the lowest (Jones and Hawtrey, 1971). In addition, the greatest percentage of total liver radioactivity occurred in the cytosol (Bresnick *et al.*, 1967; Jones and Hawtry, 1971).

However, the greatly increased persistence of DMBA in regenerating rat liver has not been previously reported. Domsky *et al.* (1963) found that unmetabolized DMBA persisted for as long as 2 weeks in newborn mice; adult mice cleared the compound much faster. They proposed that unmetabolized DMBA was biologically active as a carcinogen since newborn mice were more susceptible to DMBA induced carcinogenesis then were adults. Schmutz, Brownie and Chaudhry (1974) reported that 6 weeks after intraglandular injection of labelled DMBA 70% of the radioactivity present in rat submandibular gland homogenates could be extracted by ethyl acetate, but the extractable products were not characterized.

The reason(s) for the persistence of soluble DMBA in regenerating liver is unclear. Stoming and Bresnick (1974) reported a 32% decrease in epoxide hydrase activity 48 h after partial hepatectomy, and inhibition of this polycyclic-hydrocarbon metabolizing enzyme was correlated with enhanced 3-methylcholanthrene induced skin carcinogenesis (Burki, Stoming and Bresnick, 1974). In addition, decreased metabolism of other drugs has been reported to occur after partial hepatectomy (Van Der Decken and Hultin, 1960; Fouts, Dixon and Shultice, 1961; Henderson and Kersten, 1970; Hilton and Sartorelli, 1970) although certain drug metabolizing enzymes, including benzpyrene hydroxylase (Spencer and Fischer, 1971/1972), are inducible in actively proliferating liver (Henderson and Kersten, 1970; Hilton and Sartorelli, 1970; Chiesara, Conti and Meldolesi, 1970).

Indirect evidence suggests, however, that DMBA metabolism is altered during liver regeneration. Wheatly, Kernohan and Currie (1966) observed a lower incidence of adrenal necrosis if DMBA was administered to rats 24 h after partial hepatectomy, presumably due to the lack of formation of 7-hydroxymethyl-12-methylbenz(a)anthracene, an adrenocorticolytic metabolite. Our preliminary data (Table IV) support the concept of altered metabolism in that regenerating liver responds less than intact liver to *in vivo* DMBA administration as measured by the formation of polar metabolites of this compound by liver microsomes *in vitro*.

The relationship of DMBA persistence to carcinogenesis is also obscure. Although the active forms of unmethylated polycyclic aromatic carcinogens are thought to be epoxides, the case for methylated carcinogens like DMBA is not clear. Flesher and Sydnor (1971) proposed that a methyl carbonium ion intermediate was the ultimate carcinogen and suggested that the first step in the formation of such an intermediate is the formation of 7-hydroxymethyl-12-methylbenz(a)anthracene However, Marquardt and Heidelberger (1972) demonstrated that DMBA could transform cells lacking carcinogen metabolizing enzymes. The addition of feeder cells possessing metabolizing enzymes had no effect on the efficiency of DMBA induced transformation whereas transformation by 3-methylcholanthrene was enhanced in the presence of feeder cells. Also, although the K-region epoxide of DMBA was an active transforming agent it was slightly less active than the parent hydrocarbon (Marquardt *et al.*, 1974). More recently, Marquardt, Sapozinck and Zedeck (1974) observed that the administration of cysteamine-HCL, a free radical scavenger, reduced the incidence of DMBA induced mammary tumours as well as the incidence of fibroblast transformation *in vitro*, without inhibiting the toxic effect of DMBA. These results indicate that DMBA may act in a fundamentally different way from other polycyclic hydrocarbons.

Although the meaning of our findings is unclear at present, it is possible to envisage potential alterations in cellular control mechanisms brought about only by the long-term persistence of soluble DMBA. Alternatively, the presence of soluble DMBA might provide a reservoir of substrate for further metabolic activation steps, ultimately resulting in covalent binding to critical cellular macromolecules. Therefore, we regard it of interest that bound, and especially unbound, DMBA is found in persistently high levels in nuclei and other fractions from regenerating liver but disappears rapidly from intact liver.

Cosimo Sciotto provided many helpful comments during the course of this work. We thank Drs David Goldthwait and Oscar Sudilovsky for reviewing earlier versions of the manuscript, and Dr John R. Carter for his continuing encouragement and support. Jean Pirina and Bonnie Lou Berry provided efficient secretarial assistance.

This work was supported in part by American Cancer Society Institutional Grant No. IN57-L, and by U.S.P.H.S., Pathobiology Training Grant No. 5 T01 GM01784-08.

### REFERENCES

ALLISON, A. C. & MALLUCCI, L. (1964) Uptake of Hydrocarbon Carcinogens by Lysosomes. *Nature, Lond.*, **203**, 1024.

BLOBEL, G. & POTTER, V. R. (1966) Nuclei From Rat Liver: Isolation Method that Combines Purity with High Yield. *Science, N.Y.*, **154**, 1662.

BOLLUM, F. J. (1966) Filter Paper Disc Techniques for Assaying Radioactive Macromolecules. In *Procedures in Nucleic Acid Research.* G. L. Cantoni and D. R. Davies. New York: Harper and Row. p. 296.

BOOTH, J., KEYSELL, G. R. & SIMS, P. (1973) Formation of Glutathione Conjugates as Metabolites of 7,12 Dimethylbenz(a)anthracene by Rat Liver Homogenates. *Biochem. Pharmac.*, **22**, 1781.

BOYLAND, E. & SIMS, P. (1965) Metabolism of Polycyclic Compounds: The Metabolism of 7,12 Dimethylbenz(a)anthracene by Rat Liver Homogenates. *Biochem. J.*, **95**, 780.

BRESNICK, E., LIEBELT, R. A., STEVENSON, J. G. & MADIX, J. C. (1967) The Distribution of Radioactivity Within the Hepatic Cells after Administration of Labelled 3-Methylcholanthrene. *Cancer Res.*, **27**, 462.

BROOKES, P. & LAWLEY, P. D. (1964) Evidence for the Binding of Polynuclear Aromatic Hydrocarbons by the Nucleic Acids of Mouse Skin: Relations Between Carcinogenic Power of Hydrocarbons and their Binding to Deoxyribonucleic Acid. *Nature, Lond.*, **202**, 781.

BURKI, K., STOMING, T. A. & BRESNICK, E. (1974) Effects of an Epoxide Hydrase Inhibitor on the *in vitro* Binding of Polycyclic Hydrocarbons to DNA and on Skin Carcinogenesis. *J. natn. Cancer Inst.*, **52**, 785.

CALCUTT, G. (1958) The Distribution of Polycyclic Hydrocarbons Within the Cells of Some Mouse and Rat Tissues. *Br. J. Cancer*, **12**, 149.

CALCUTT, G. & PAYNE, S. (1954*a*) The Intracellular Metabolism of 3,4 Benzpyrene: Sites of Oxidation in Mouse Liver. *Br. J. Cancer*, **8**, 554.

CALCUTT, G. & PAYNE, S. (1954*b*) The Intracellular Metabolism of 3,4 Benzpyrene: Further Examination of the Supernatant Fraction from Mouse Liver. *Br. J. Cancer*, **8**, 561.

CALCUTT, G. & PAYNE, S. (1954*c*) The Intracellular Metabolism of 3,4, Benzpyrene: Benzpyrene Metabolites from Rats and Their Sites of Formation in Rat Liver. *Br. J. Cancer*, **8**, 710.

CERIOTTI, G. (1952) A Microchemical Determination of Desoxyribonucleic Acid. *J. biol. Chem.*, **198**, 297.

CHIESARA, E., CONTI, F. & MELDOLESI, J. (1970) Influence of Partial Hepatectomy on the Induction of Liver Microsomal Drug Metabolizing Enzymes Produced by Phenobarbital. *Lab. Invest.*, **22**, 329.

DEPIERRE, J. W., MORON, M. S., JOHANNESEN, K. A. M. & ERNSTER, L. (1975) A Reliable, Sensitive and Convenient Radioactive Assay for Benzpyrene Monooxygenase. *Anal. Biochem.*, **63**, 470.

DIAMOND, L., DEFENDI, V. & BROOKES, P. (1967) The Interaction of 7,12 Dimethylbenz(a)anthracene with Cells Sensitive and Resistant to Toxicity Induced by this Carcinogen. *Cancer Res.*, **27**, 890.

DOMSKY, I., LIJINSKY, W., SPENCER, K. & SHUBIK, P. (1963) Rate of Metabolism of 9,10 Dimethyl 1,2 Benzanthracene in Newborn and Adult Mice. *Proc. Soc. exp. Biol. Med.*, **113**, 110.

FLESHER, J. W. & SYDNOR, K. L. (1971) Carcinogenicity of Derivitives of 7,12-Dimethylbenz(a)-anthracene. *Cancer Res.*, **31**, 1951.

FOUTS, J. R., DIXON, R. L. & SHULTICE, R. W. (1961) The Metabolism of Drugs by Regenerating Liver. *Biochem. Pharmac.*, **7**, 265.

GOSHMAN, L. M. & HEIDELBERGER, C. (1967) Binding of Tritium Labelled Polycyclic Hydrocarbons to DNA of Mouse Skin. *Cancer Res.*, **27**, 1678.

HENDERSON, P. T. & KERSTEN, K. J. (1970) Metabolism of Drugs during Rat Liver Regeneration. *Biochem. Pharmac.*, **19**, 2343.

HIGGINS, G. M. & ANDERSON, R. M. (1931) Experimental Pathology of Liver. I. Restoration of Liver of White Rat Following Partial Surgical Removal. *Archs Path.*, **12**, 186

HILTON, J. & SARTORELLI, A. C. (1970) Induction of Microsomal Drug Metabolizing Enzymes in Regenerating Liver. *Adv. Enzyme Res.*, **8**, 153.

IYPE, P. T. & OCKEY, C. H. (1971/1972) Ultrastructural Localization of Tritiated DMBA bound to Malignant and Non-malignant Cell Lines. *Chem.-Biol. Interact.*, **4**, 71.

JANSS, D. H., MOON, R. C. & IRVING, C. C. (1972) The Binding of 7,12 Dimethylbenz(a)anthracene to Mammary Parenchyma DNA and Protein *in vivo*. *Cancer Res.*, **32**, 254.

JELLINCK, P. H. & GOUDY, B. (1967) Effect of Pretreatment with Polycyclic Hydrocarbons on the Metabolism of DMBA-12-14C by Rat Liver and Other Tissues. *Biochem. Pharmac.*, **16**, 131.

JELLINCK, P. H., SMITH, G. & FLETCHER, R. (1970) Nature of the Water Soluble Metabolites of 7,12 Dimethylbenz(a)anthracene formed by Liver Microsomes of Normal and 3-Methylcholanthrene treated rats. *Cancer Res.*, **30**., 1715.

JONES, P. A. & HAWTREY, A. O. (1971) Studies on the Binding and Distribution of Radioactively Labelled 3-Methylcholanthrene in Subcellular Fractions of Rat Liver. *Br. J. Cancer*, **25**, 845.

JUHN, S. K. & PRODI, G. (1965) The effect of 7,12 Dimethylbenz(a)anthracene on the Incorporation of $^{3}$H-Thymidine into DNA in Normal and Regenerating Liver. *Experientia*, **12**, 473.

KUROKI, T. & HEIDELBERGER, C. (1971) The Binding of Polycyclic Aromatic Hydrocarbons to DNA, RNA, and Protein of Transformable Cells in Culture. *Cancer Res.*. **31**, 2168.

LEES, M. B. & PAXMAN, S. (1972) Modification of the Lowry Procedure for the Analysis of Proteolipid Protein. *Analyt. Biochem.*, **47**, 184.

LEVINE, W. G. (1974) Hepatic Uptake, Metabolism and Biliary Excretion of 7,12 Dimethylbenz(a)anthracene in the Rat. *Drug metab. Disp.*, **2**, 169.

LOWRY, O. H.., ROSEBROUGH, N. J., FARR, A. L. & RANDALL, R. J. (1951) Protein Measurement with the Folin Phenol Reagent. *J. biol. Chem.*, **193**,265.

MARQUARDT, H., BENDICH, A., PHILIPS, F. S. & HOFFMANN, D. (1971) Binding of (G-$^{3}$H)-7,12 Dimethylbenz(a)anthracene to DNA of Normal and of Rapidly Dividing Hepatic Cells of Rats. *Chem.-Biol. Interact.*, **3**, 1.

MARQUARDT, H. & HEIDELBERGER, C. (1972) Influence of "Feeder Cells" and Inducers and Inhibitors of Microsomal Mixed Function Oxidases on Hydrocarbon Induced Malignant Transformation of Cells Derived from C3H Mouse Prostate. *Cancer Res.*, **23**, 721.

MARQUARDT, H. & PHILIPS, F. S. (1970) The Effects of 7,12 Dimethylbenz(a)anthracene on the Synthesis of Nucleic Acids in Rapidly Dividing Hepatic Cells in Rats. *Cancer Res.*, **30**, 2000.

MARQUARDT, H., PHILIPS, F. S. & BENDICH, A. (1972) DNA Binding and Inhibition of DNA Synthesis after 7,12 Dimethylbenz(a)anthracene Administered During the Early Pre-replicative Phase in Regenerating Rat Liver. *Cancer Res.*, **32**, 1810.

MARQUARDT, H., SAPOZINK, M. D. & ZEDECK, M. S. (1974) Inhibition by Cysteamine HCl of Oncogenesis Induced by 7,12-Dimethylbenz(a)anthracene without affecting Toxicity. *Cancer Res.* **34**, 3387.

MARQUARDT, H., SODERGREN, J. E., SIMS, P. & GROVER, P. L. (1974) Malignant Transformation *in vitro* of Mouse Fibroblasts by 7,12 Dimethylbenz(a)anthracene and 7-Hydroxymethyl-12-methylbenz(a)anthracene and by their K-region Derivatives. *Int. J. Cancer*, **13**, 304.

MARQUARDT, H., STERNBERG, S. S. & PHILIPS, F. S. (1970) 7,12 Dimethylbenz(a)anthracene and Hepatic Neoplasia in Regenerating Rat Liver. *Chem.-Biol. Interact.*, **2**, 401.

NAKAI, T. & SHUBIK, P. (1964) Autoradiographic Localization of Tissue Bound Tritiated 7,12-DMBA in Mouse Skin 24 and 48 Hours After Single Application. *J. natn. Cancer Inst.*, **33**, 887.

NEBERT, D. W. & GIELEN, J. E. (1972) Genetic Regulation of Aryl Hydrocarbon Hydroxylase Induction in the Mouse. *Fedn Proc.*, **31**, 1315.

POUND, A. W. (1968) Carcinogenesis and Cell Proliferation. *N.Z. med. J.*, **67**, 88.

PRODI, G. ROCCHI, P. & GRILLI, S. (1970) Binding of 7,12 Dimethylbenz(a)anthracene and 1,2 Benz(a)anthracene to Nucleic Acids and Proteins of Organs in Rats. *Cancer Res.*, **30**, 1020.

ROGAN, E. G. & CAVALIERI, E. (1974) 3-Methylcholanthrene Inducible Binding of Aromatic Hydrocarbons to DNA in Purified Rat Liver Nuclei. *Biochem. Biophys. Res. Commun.*, **58**, 1119.

SCHMUTZ, J. A., BROWNIE, A. C. & CHAUDHRY, A. P. (1974) 7,12 Dimethylbenz(a)anthracene

Retention in the Rat Submandibular Gland Following Intraglandular Injection. *Cancer Res.*, **34**, 576.

SCHWEPPE, J. S., KOT, E. & JUNGMAN, R. A. (1971) Effects of Gonadectomy on the Uptake of Polycyclic Hydrocarbons by Rat Liver Nuclei, DNA and Histones. *Proc. Soc. exp. Biol. Med.*, **138**, 167.

SPENCER, T. & FISHER, P. W. F. (1971/1972) The Induction of Microsomal Hydroxylases in Regenerating Rat Liver. *Chem.-Biol. Interact.*, **4**, 41.

STOMING, T. A. & BRESNICK, E. (1974) Hepatic Epoxide Hydrase in Neonatal and Partially Hepatectomized Rats. *Cancer Res.*, **34**, 2810.

T'SO, P. O. P. & LU, P. (1964) Interaction of Nucleic Acids II. Chemical Linkage of the Carcinogen 3,4 Benzpyrene to DNA Induced by Photoradiation. *Proc. natn. Acad. Sci. U.S.A.*, **51**, 272.

VAN DER DECKEN, A. & HULTIN, T. (1960) The Enzymatic Composition of Rat Liver Microsomes During Liver Regeneration. *Expl Cell Res.*, **19**, 591.

WHEATLEY, D. N., KERNOHAN, I. R. & CURRIE, A. R. (1966) Liver Injury and the Prevention of Massive Adrenal Necrosis from 9,10-Dimethyl-1,2-Benzanthracene in Rats. *Nature, Lond.*, **211**, 387.